# Surgical fat removal exacerbates metabolic disorders but not atherogenesis in LDLR^−/−^ mice fed on high-fat diet

**DOI:** 10.1038/s41598-019-54392-8

**Published:** 2019-11-28

**Authors:** Lin Liu, Chenxi Liang, Xiaowei Wang, Xiayu Ding, Yingjing Lu, Jinghui Dong, Mei Han, Hongyuan Yang, Jiawei Liao, Mingming Gao

**Affiliations:** 1 Laboratory of Lipid Metabolism, Institute of Basic Medicine, Hebei Medical University, Shijiazhuang, Hebei 050017 China; 2 Department of Physiology, Hebei Medical University, Shijiazhuang, Hebei 050017 China; 3 Department of Biochemistry and Molecular Biology, College of Basic Medicine, Key Laboratory of Medical Biotechnology of Hebei Province, Hebei Medical University, Shijiazhuang, Hebei 050017 China; 4 School of Biotechnology and Biomolecular Sciences, The University of New South Wales, Sydney, NSW 2052 Australia; 5 Department of Cardiology, Institute of Cardiovascular Diseases, First Affiliated Hospital of Dalian Medical University, Dalian, Liaoning 116011 China

**Keywords:** Fats, Acute coronary syndromes

## Abstract

Lipodystrophy is a severe adipose dysfunction that can be classified as congenital or acquired lipodystrophy, in term of the etiology. Previous knowledge about the metabolic disorders and cardiovascular consequences were mostly obtained from lipodystrophic mice with genetic defects. To completely rule out the genetic influence, we established a mouse model of acquired generalized lipodystrophy by surgical removal of multiple fat depots, including subcutaneous fat in the inguinal, visceral fat in the epididymis and brown fat in the scapula, in atherosclerosis-prone LDLR^−/−^ mice which were fed with a high-fat diet (HFD). It was observed that fat removal increased diet-induced hyperlipidemia, especially hypercholesteremia, as early as 2 weeks after HFD and till the end of HFD feeding. After 12 weeks on the HFD, the residual fats of fat-removed mice were found expanded. Although fat removal aggravated diet-induced lipid deposition in the liver and systemic insulin resistance, there was no significant difference in atherogenesis in fat-removed mice compared with sham-operated control mice. Acquired generalized lipodystrophy by surgical fat removal promoted metabolic disorders but not atherogenesis in LDLR^−/−^ mice fed on HFD.

## Introduction

The adipose tissue is a very important energy storage and endocrine organ, whose dysfunction can disrupt systemic metabolic homeostasis and cause serious consequences, such as dyslipidemia, fatty liver, insulin resistance as well as atherosclerotic cardiovascular diseases, etc. Both increased adiposity (obesity) and decreased adiposity (lipodystrophy) can lead to severe adipose dysfunction^1,2^. However, with the striking and ongoing increase of obese population, the majority of research focuses have been devoted to obesity and subsequent metabolic and cardiovascular research^3–5^.

Lipodystrophy, in term of the etiology, can be classified as congenital or acquired lipodystrophy. The former is caused by genetic defects or some syndromes disrupting adipogenesis and differentiation, lipid droplet formation as well as adipocyte apoptosis, etc. According to the degree of fat loss, congenital lipodystrophy can be divided into two categories: congenital generalized lipodystrophy (CGL) (causative gene: AGPAT2, BSCL2, CAVI, PTRF) and familial partial lipodystrophy (FPLD) (causative gene: PPARG, LMNA, ZMPSTE24, AKT2, CIDEC, etc.)^6^. Conversely, acquired lipodystrophy does not have a direct genetic defect but can be induced by drugs, autoimmunity or unknown (idiopathic) causes, which further divided into four types: acquired generalized lipodystrophy (AGL), acquired partial lipodystrophy (APL), lipodystrophy in human immunodeficiency virus (HIV) infected patients and local lipodystrophy^7,8^. No matter what type of lipodystrophy, the correlation of this disease to metabolic disorders and cardiovascular consequences is largely unknown, possibly due to the relatively small proportion of patients or the lack of suitable animal models. Of the limited literatures about lipodystrophic patients, it has been reported that CGL and AGL patients suffered severe dyslipidemia (mainly hypertriglyceridemia) and early diabetes due to extreme fat loss; however, only a small proportion (4 in 80) of these patients developed early onset coronary heart disease^9^. As to the HIV patients, those on protease inhibitor cocktail treatment suffered from both severe fat loss in the lambs and face and severe fat increase in the abdomen and back, therefore the observed severe dyslipidemia and insulin resistance and a significantly higher risk of developing coronary heart disease in these patients could not be simply attributed to the increased adiposity in the abdomen or the decreased adiposity in the lambs and face^10^.

Previously, we generated a generalized lipodystrophic mouse model with Seipin deficiency (causative gene of CGL2), which showed systemic fat loss, severe fatty liver and insulin resistance^11^. When crossed with atherosclerosis (As)-prone low-density lipoprotein receptor (LDLR) knockout (LDLR^−/−^) or apolipoprotein E (ApoE) knockout (ApoE^−/−^) mice, Seipin deficiency significantly aggravated As^12,13^. Moreover, Seipin and LDLR double knockout (Seipin^−/−^LDLR^−/−^) mice even developed extremely hypertriglyceridemia and hypercholesterolemia after high-fat diet (HFD) feeding (Triglyceride: ~6000 mg/dL; Cholesterol: ~8000 mg/dL). Rosiglitazone, an agonist of PPARg, a key nuclear receptor for adipogenesis, could significantly reduce plasma triglyceride and cholesterol levels, restore partial adipose tissue, and inhibit As^12^. Another mouse model of congenital lipodystrophy, Fld mice (Lipin mutation) were of the traits of neonatal fatty liver as well as hypertriglyceridemia that resolved when they weaned^14^. Adult Fld mice lost about 80% of total adipose tissue and presented with insulin resistance^15^. Although there were lower plasma atherogenic VLDL/LDL level and higher anti-atherosclerotic HDL level in Fld mice than those of wild-type controls, the atherogenesis in Fld mice was accelerated^15^. However, whether the observed metabolic disorders and aggravated As in these two models are directly caused by fat loss, or gene-dependent has not been fully defined.

To completely rule out the genetic influence in fat loss, we established a mouse model of acquired generalized lipodystrophy by surgical removal of multiple fat depots, including subcutaneous fat in the inguinal, visceral fat in the epididymis and brown fat in the scapula, in As-prone LDLR^−/−^ mice. Unlike congenital generalized lipodystrophy, acquired generalized lipodystrophy by surgical fat removal did not promote atherogenesis, in spite of aggravated hyperlipidemia, fatty liver and insulin resistance.

## Results

### Surgical fat removal aggravated diet-induced hyperlipidemia in LDLR^−/−^ mice

**Figure 1:**
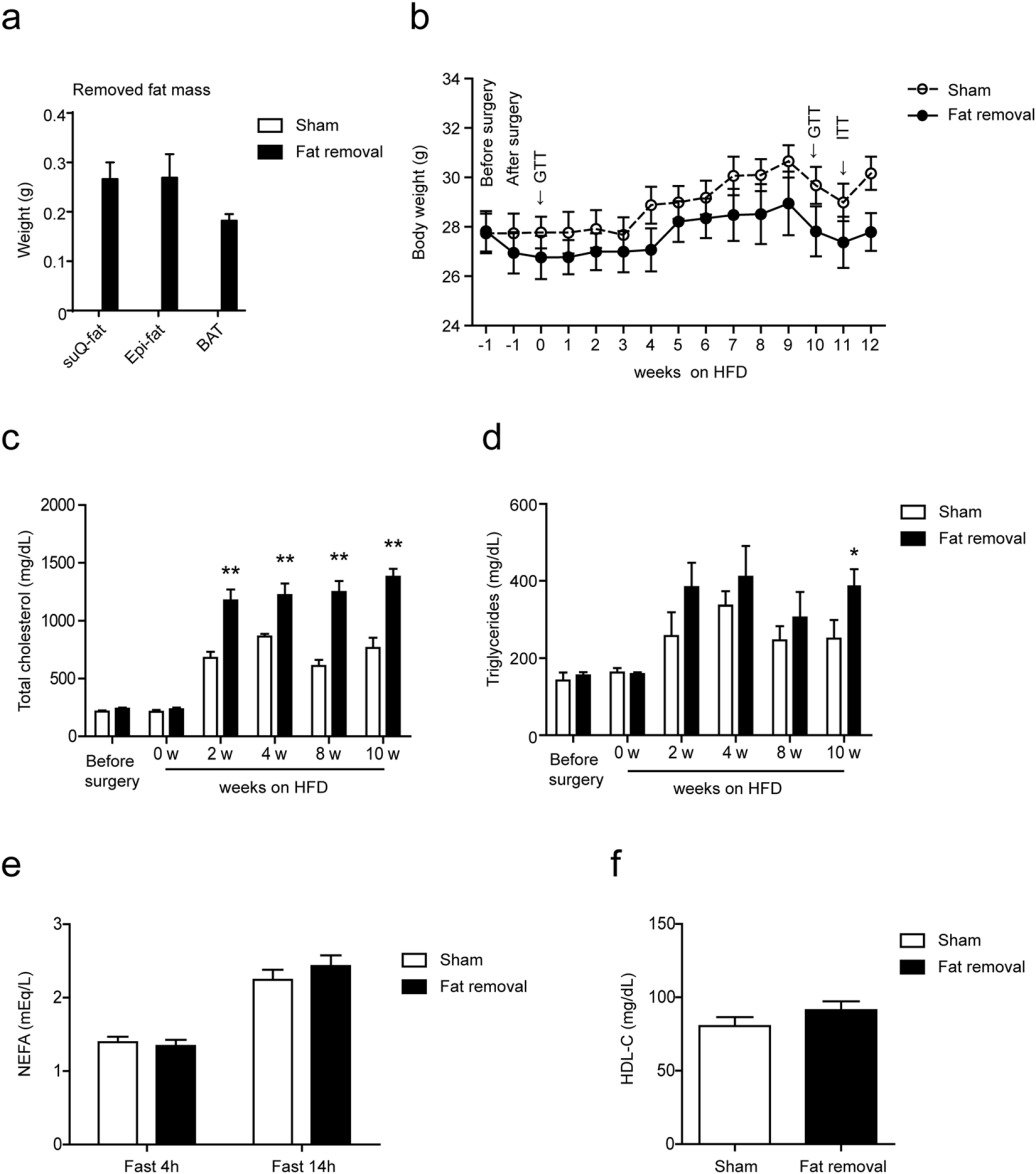
Aggravated diet-induced hyperlipidemia in the fat-removed LDLR^−/−^ mice. (**a**) Weight of removed fats during the fat-removal surgery. (**b**) Body weight before the fat removal surgery and during HFD feeding. (**c**,**d**) Plasma total cholesterol and triglyceride levels before the fat removal surgery and during HFD feeding. (**e**) Plasma non-esterified fatty acid levels after 10 weeks on the HFD feeding. (**f**) Plasma high-density lipoprotein cholesterol level after 10 weeks on the HFD feeding, n = 8–9 for each group, *P < 0.05, **P < 0.01. suQ-fat: subcutaneous fat; Epi-fat: epididymal fat; and BAT: brown adipose tissue; GTT: Glucose tolerance test; ITT: Insulin tolerance test.

The adipose tissue, according to the appearance and function, are usually divided into white adipose and brown adipose. The former is further divided into subcutaneous fat and visceral fat. While the brown adipose can be mainly found in the scapula of an adult mouse, both subcutaneous fat and visceral fat are distributed in multiple depots. In our acquired generalized lipodystrophy model, subcutaneous fat in the inguinal, visceral fat in the epididymis and brown fat in the scapula were chosen to be surgically removed, while other fat depots were retained. The removed fats were weighted and shown in Fig. 1a. The removed fats accounted for about 20–40% of the total fats. 1 week after the surgery, all mice were subjected with a high-fat diet (HFD) feeding to induce metabolic disorders and atherogenesis. Weight gain during HFD feeding were recorded every week. As what was shown in Fig. 1b, the body weight of the fat-removed mice was consistently lower than that of the sham-operated control mice, although no significance was reached. Although the levels of plasma total cholesterol (TC) and triglycerides (TG) were not of difference between the fat-removed mice and sham-operated control mice before the fat removal surgery, the plasma total cholesterol level in the fat-removed mice began to be almost double of that in the sham-operated mice since 2 weeks on the HFD and all the way to the end of HFD feeding (Fig. 1c), while the triglycerides increase in the fat-removed mice was not so significant as that of the total cholesterol increase (Fig. 1d). The levels of plasma non-esterified fatty acids (NEFA) or high-density lipoprotein cholesterol (HDL-C), however, did not change much after fat removal (Fig. 1e,f). Our data demonstrated that acquired generalized lipodystrophy by surgical fat removal aggravated diet-induced hyperlipidemia in the fat-removed LDLR^−/−^ mice.

### Surgical fat removal induced compensatory adaptions of the residual fats in LDLR^−/−^ mice

**Figure 2:**
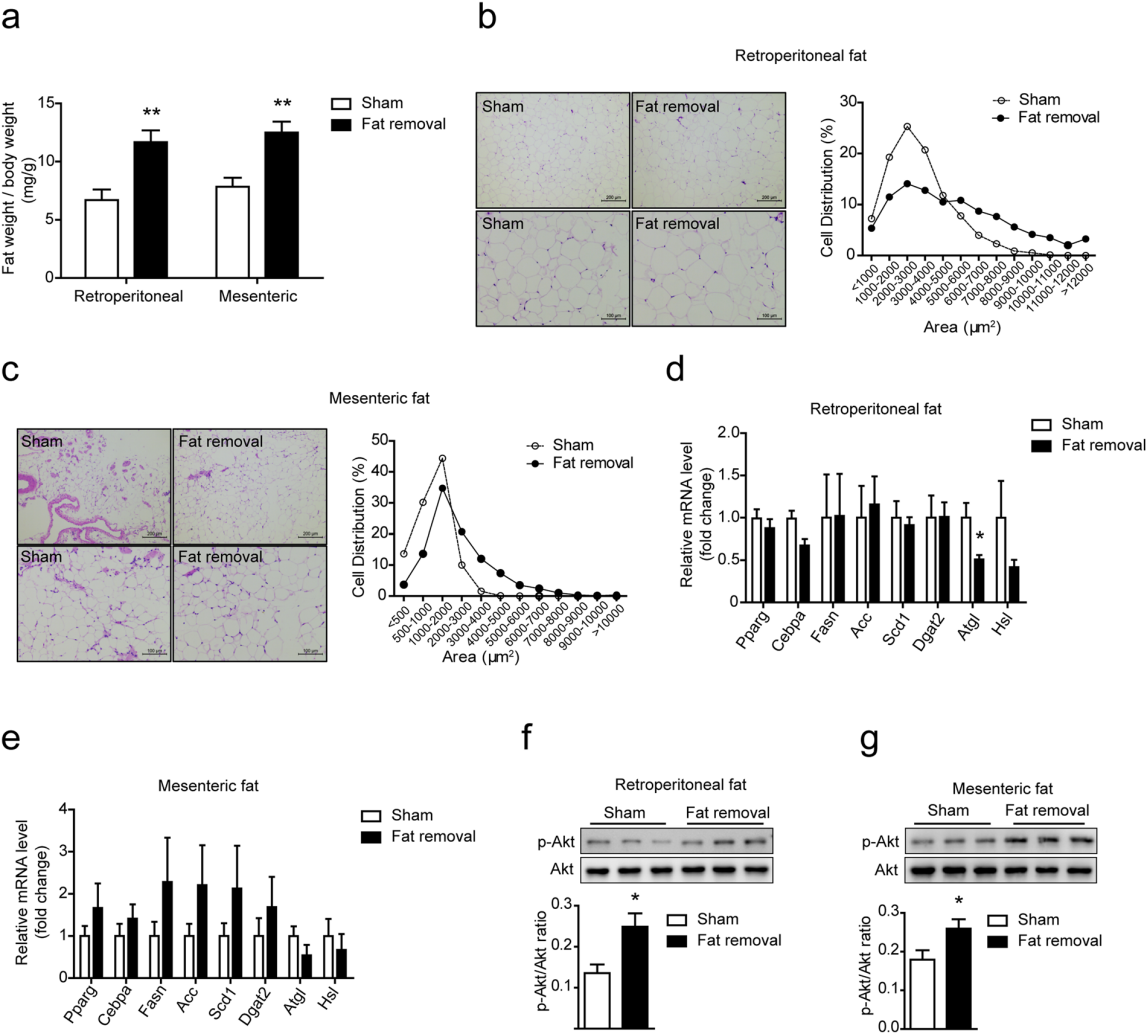
Compensatory adaptions of the residual fats in the fat-removed LDLR^−/−^ mice. (**a**) Weight of retroperitoneal and mesenteric fat, n = 8–9 for each group, **P < 0.01. (**b**,**c**) H&E staining and adipocyte size distribution of retroperitoneal and mesenteric fat. Bar = 100 μm (bottom) or 200 μm (top). Each distribution was obtained from four mice and at least 300 adipocytes in one mouse in H&E staining. (**d**,**e**) mRNA expr**e**ssion of genes related with adipogenesis, lipid synthesis and lipolysis in retroperitoneal and mesenteric fat. n = 6 for each group, *P < 0.05. **(f,g)** Western-blot analysis of p-Akt (Ser473) and total Akt in the retroperitoneal **(f)** and mesenteric **(g)** fat, n = 6 for each group, *P < 0.05.

12 weeks after fed on the HFD, all the experimental mice were sacrificed. Residual fats were first examined for potential compensatory adaptions. We found that the retroperitoneal fat and mesenteric fat in the fat-removed mice were significantly expanded (Fig. 2a). Hematoxylin/eosin staining (H&E staining) and quantitative results of adipocyte area of the two depots showed slightly increased adipocyte size (Fig. 2b,c). Expression of genes associated with adipogenesis (*Pparg and Cebpa*), lipid synthesis (*Fasn, Acc, Scd1, Dgat2*) and lipolysis (*Atgl and Hsl*) were investigated by real-time PCR. We found that the mRNA level of Atgl was decreased in the retroperitoneal fat of the fat-removed mice (Fig. 2d,e). We further examinated the expression of Akt phosphorylation and activation in the two fat depots by western-blot analysis and the results showed that phosphorylated-Akt (p-Akt) was significantly up-regulated in the retroperitoneal fat and mesenteric fat of fat-removed mice, suggesting that cellular proliferation of the residual fats might be increased and also insulin sensitivity might be increased (Fig. 2f,g). Together, these data demonstrated that acquired generalized lipodystrophy by surgical fat removal induced compensatory adaptions of the residual fat depots in the fat-removed LDLR^−/−^ mice.

### Surgical fat removal aggravated fatty liver in LDLR^−/−^ mice

**Figure 3:**
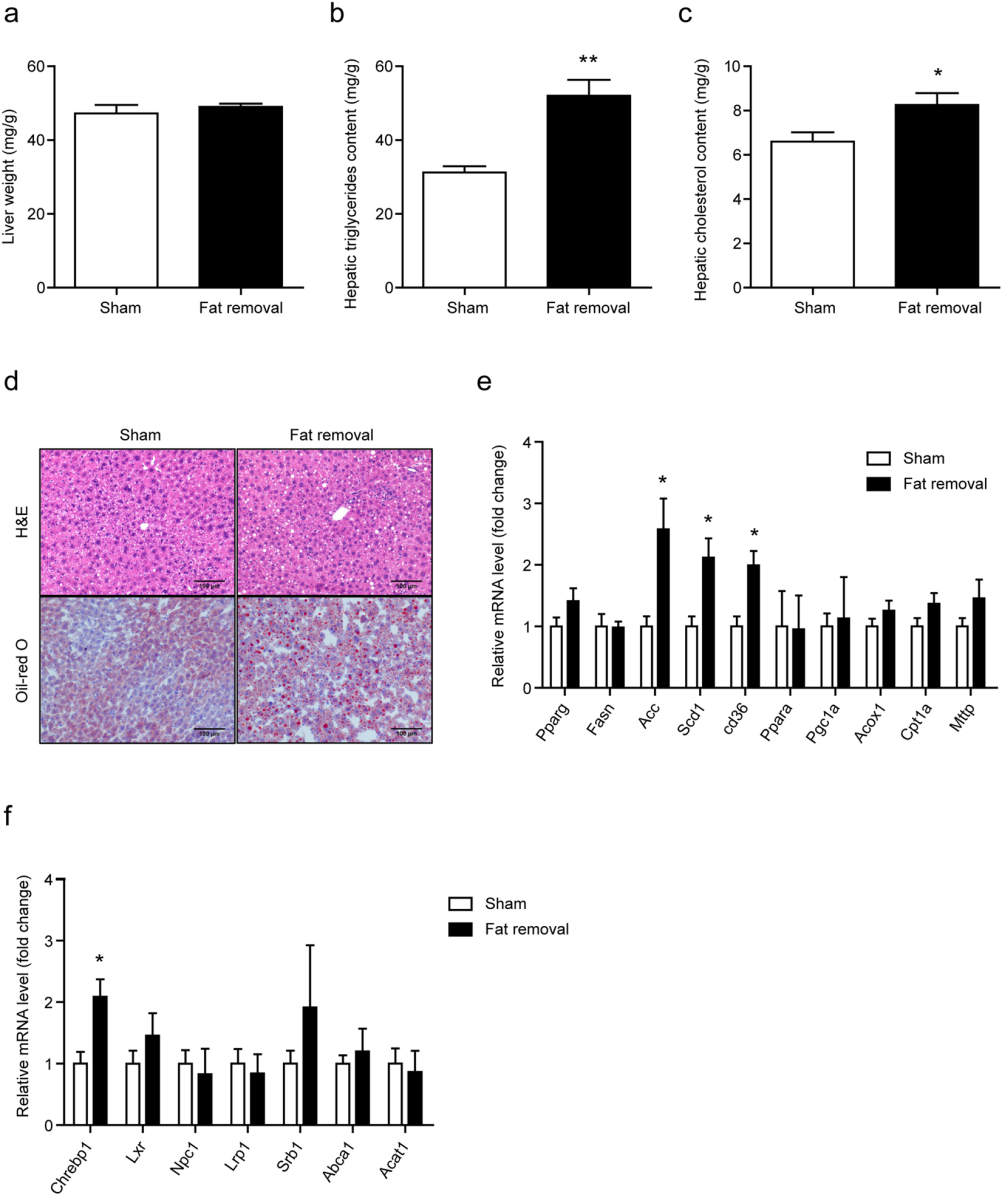
Aggravated fat deposition in the liver of the fat-removed LDLR^−/−^ mice. (**a**) Liver weight. (**b**,**c**) Liver triglyceride and total cholesterol content, n = 8–9 per group, **P < 0.01. (**d**) H&E (top) and Oil-red O (bottom) staining of the liver. Bar = 100 μm. (**e**,**f**) mRNA expression of genes related with triglyceride and cholesterol metabolism in the liver. n = 6 for each group, *P < 0.05.

We then explored lipid deposition in the liver in the fat-removed mice fed on the HFD. We found that although there was no significant changes in the liver weight (Fig. 3a), the hepatic triglyceride and cholesterol content, determined by lipid extraction, in the fat-removed mice were significantly increased compared with the sham-operated control mice (Fig. 3b,c). Further analysis using H&E and Oil-red O staining verified more lipid deposition in the liver (Fig. 3d). We then detected the expression of genes related with triglyceride and cholesterol metabolism by real-time PCR. It was shown that the mRNA levels of triglyceride synthesis related genes, including *Acc, Scd1* and *Cd36*, together with *Chrebp*, which mediated the conversion of carbohydrates to lipids, were significantly up-regulated in fat-removed mice, suggesting that lipogenesis was increased in the fat-removed mice (Fig. 3e,f). These data demonstrated that acquired generalized lipodystrophy by surgical fat removal aggravated hepatic lipid deposition in the fat-removed LDLR^−/−^ mice.

### Surgical fat removal aggravated insulin resistance in LDLR^−/−^ mice

**Figure 4:**
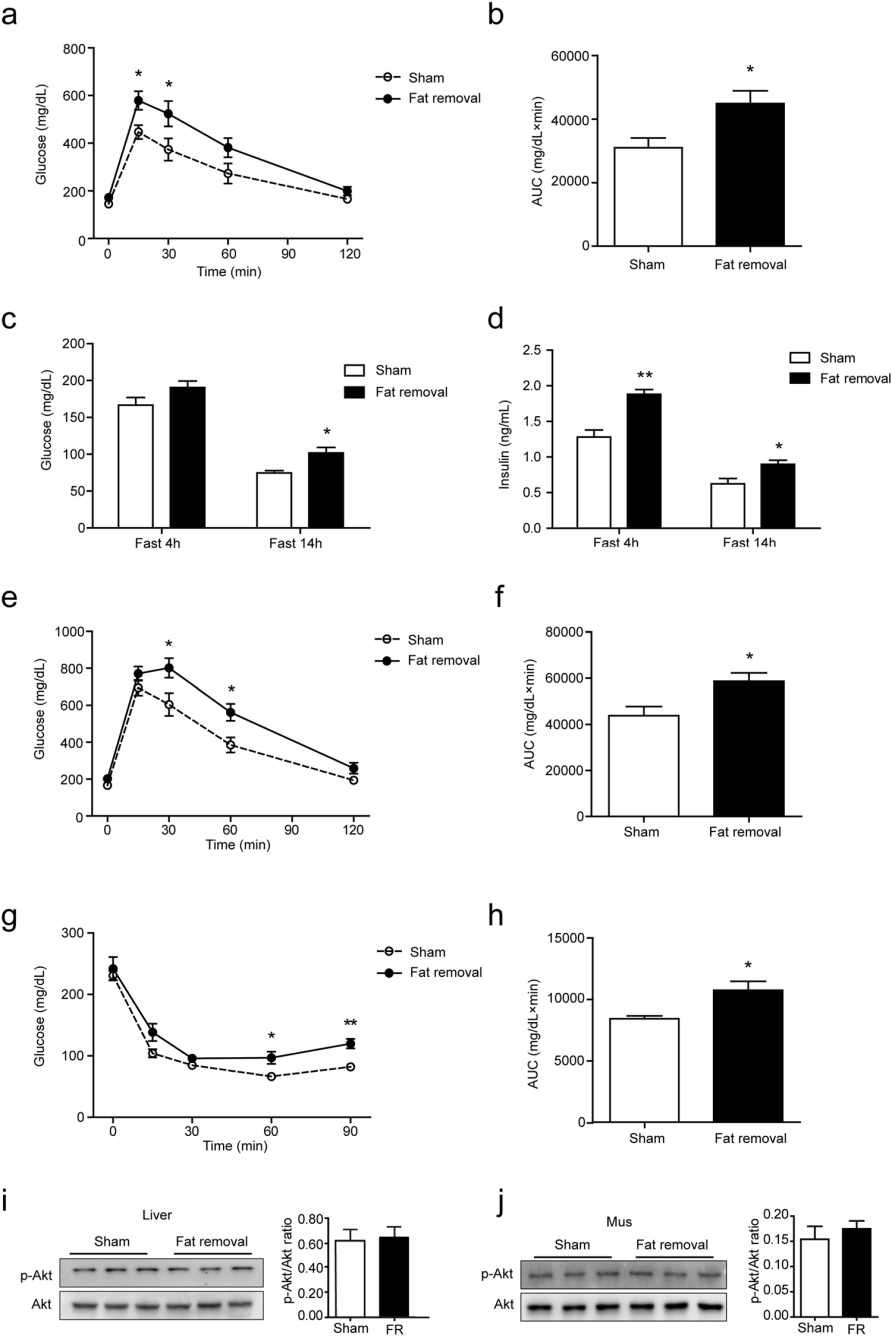
Aggravated insulin resistance in the fat-removed LDLR^−/−^ mice. (**a**) Glucose tolerance test (GTT) after 1 week’s recovery from the fat removal surgery. (**b**) Area under curve of figure (**a**). (**c**) Plasma glucose level after 10 weeks on the HFD feeding. (**d**) Plasma insulin level after 10 weeks on the HFD feeding. (**e**) Glucose tolerance test (GTT) after 10 weeks on the HFD feeding. (**f**) Area under curve of figure (**e**). (**g**) Insulin tolerance test (ITT) after 11 weeks on the HFD feeding. (**h**) Area under curve of figure (**g**), n = 8–9 for each group, *P < 0.05, **P < 0.01. (**I**,**j**) Western-blot analysis of p-Akt (Ser473) and total Akt in the liver **(i)** and skeletal muscle **(j)**, n = 6 for each group. FR: Fat removal.

Next, we explored the insulin sensitivity in the fat-removed mice fed on the HFD. By glucose tolerance test, we found that the fat-removed mice developed increased glucose intolerance as early as 1 week after the fat removal surgery while the mice were still subjected to the chow diet (Fig. 4a,b). After fed on the HFD, a significant increase of plasma glucose upon overnight fasting was observed in the fat-removed mice, as compared to the sham-operated control mice, although the plasma glucose level during a short-time (4 hours) fasting did not change much (Fig. 4c). Plasma insulin level in the fat-removed mice was also significantly increased (Fig. 4d). Moreover, glucose tolerance (Fig. 4e,f) as well as insulin tolerance (Fig. 4g,h) tests further confirmed the increase of glucose intolerance and insulin insensitivity after fat removal. Akt has been proved as a downstream molecule of insulin signaling pathway. We observed that the p-Akt levels in the insulin-sensitive liver and skeletal muscle did not show significant changes (Fig. 4i,j), however, the phosphorylation and activation of Akt in the residual fats, especially in the retroperitoneal fat and mesenteric fat, were significantly up-regulated (Fig. 2f,g), although the increased insulin sensitization failed to compensate for the systemic insulin resistance caused by the removal of the three fat depots. Together, these data demonstrated that acquired generalized lipodystrophy by surgical fat removal aggravated diet-induced insulin resistance in the fat-removed LDLR^−/−^ mice.

### Surgical fat removal did not promote atherogenesis in LDLR^−/−^ mice

**Figure 5:**
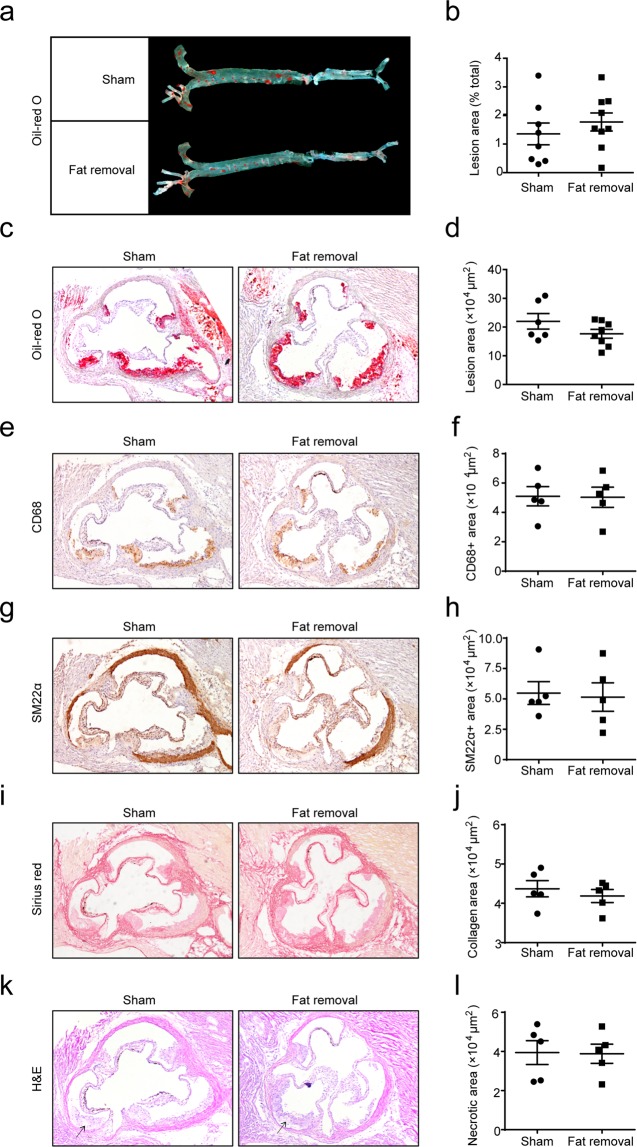
No altered atherogenesis in the fat-removed LDLR^−/−^ mice. (**a**,**b**) Oil-red O staining of the *en face* aortae and quantitative analysis of the aortic lesion area, n = 8–9 for each group. (**c**,**d**) Oil-red O staining of the aortic root and quantitative analysis of the aortic root lesion area, n = 6–8 for each group. (**e**,**f**) CD68 immunochemical staining of the aortic root and quantitative analysis of the CD68^+^ macrophage content in the lesions, n = 5 for each group. (**g**,**h**) SM22α immunochemical staining of the aortic root and quantitative analysis of the SM22α^+^ smooth muscle cell content in the lesions, n = 5 for each group. (**I**,**j**) Sirius Red staining of the aortic root and quantitative analysis of the collagen content in the lesions, n = 5 for each group. (**k**,**l**) H&E staining of the aortic root and quantitative analysis of the necrotic core area in the lesions. The arrows indicated the necrotic core, n = 5 for each group.

Finally, we explored the atherogenesis in the fat-removed mice fed on the HFD. Unexpectedly, no increase of As area, measured by Oil-red O staining, was found in the aorta as well as the aortic root of the fat-removed mice after 12 weeks on the HFD, as compared with the sham-operated control mice (Fig. 5a–d). Moreover, no significant alterations of the lesion components, including lesional macrophages, visualized by CD68 immunochemical staining (Fig. 5e,f), lesional smooth muscle cells, visualized by SM22α immunochemical staining (Fig. 5g,h), or collagen content, visualized by Sirius red staining (Fig. 5i,j), were observed in the fat-removed mice. Formation of the necrotic core (designated as eosin-negative acellular areas in H&E staining), a hallmark of advanced lesions and parameter of plaque instability, was also not altered in the fat-removed mice (Fig. 5k,l). These data suggested that acquired generalized lipodystrophy by surgical fat removal did not promote atherogenesis, in spite of aggravated hypercholesterolemia, fatty liver and insulin resistance, in the fat-removed LDLR^−/−^ mice.

## Discussion

In the present study, we generated an acquired generalized lipodystrophic mouse model in LDLR^−/−^ mice by surgical removal of multiple fat depots, including subcutaneous fat in the inguinal, visceral fat in the epididymis and brown fat in the scapula, and explored the metabolic disorders and subsequent atherogenesis on HFD feeding. We found that (1) Increased hyperlipidemia, especially hypercholesterolemia, was observed during HFD feeding in the fat-removed mice as compared with the sham-operated mice. (2) The residual retroperitoneal fat and mesenteric fat in the fat-removed mice had a compensatory expansion. (3) The liver of the fat-removed mice accumulated more lipids. (4) The fat-removed mice developed increased glucose intolerance and insulin resistance as early as 7 days on the HFD feeding. (5) Atherogenesis in the fat-removed mice was not exacerbated, in spite of the increased metabolic disorders described above.

Previous studies have indicated that the adipose tissue might contribute to the clearance of plasma cholesterol^16^. When mice were fed on HFD, clearance of plasma cholesterol by liver as well as adipose tissue was impaired, resulting in cholesterol accumulation in the circulation. Fat removal further decreased the adipose clearance of plasma cholesterol, therefore contributed to the observed increased hypercholesteremia. Interestingly, in the fat-removed group, residual retroperitoneal fat and mesenteric fat were compensatory expanded due to increased Akt phosphorylation and lipogenesis and decreased lipolysis. Our data suggested that removal of partial fat could induce expansion of residual fats and compensatory store more lipid in these depots. Increase of Akt phosphorylation also indicated that insulin signaling pathway in the residual adipose tissues was possibly more active and might improve systemic metabolism^17,18^.

Adipose tissue is the main storage organ for triglycerides when there is excess energy, and releases energy during fasting or starvation^19^. Loss of adipose as in lipodystrophy leads to the disorder of triglyceride storage and ectopic storage in the liver, muscle, heart and vessels, leading to fatty liver, insulin resistance and cardiovascular diseases, etc^9,20^. In the fat-removed mice, lipid deposition, especially triglycerides deposition, was significantly increased. Adipose tissue can also store body cholesterol^16,21^. In the fat-removed mice, hepatic cholesterol accumulation was also significantly increased, suggesting that in the absence of LDLR and the insufficiency of adipose tissue, increased dietary fat intake could also lead to additional cholesterol deposition in the liver.

It has been illustrated that adipose tissue was closely related to insulin sensitivity. The subcutaneous fat, by secreting cytokines such as adiponectin, could protect against fat cumulation in the visceral fat, liver as well as skeletal muscle. Therefore, insulin sensitivity is increased. In contrast, the visceral fat, prone to secrete IL-6, TNFα and other inflammatory factors, promotes ectopic lipid accumulation and results in reducing insulin sensitivity^22,23^. In HFD-induced obese mice, visceral fat removal increased insulin sensitivity, while removal of the subcutaneous fat led to the opposite^24,25^. However, no literature focused on mice being removed subcutaneous fat and visceral fat together, let alone the brown adipose tissue that is mainly responsible for thermogenesis. Therefore, our study for the first time explored the metabolic regulation in the context of adipose dysfunction of multiple depots.

Although metabolic disorders were increased in the fat-removed mice compared with those in the sham-operated mice, the atherogenesis was not exacerbated. Unlike congenital generalized lipodystrophy in Seipin^−/−^LDLR^−/−^ mice as we previously reported or in other mouse models with genetic defects^12,15^, where all the fat depots were ill-functioned, there are still residual fat depots with normal function in our fat-removed mice. The residual fat could possibly maintain certain compensatory metabolic regulations such that the increase of plasma lipids and hepatic lipid deposition were not as significant as those observed in Seipin^−/−^LDLR^−/−^ mice fed on the same HFD. Also, the peri-vascular adipose tissue in our fat-removed mice was retained. Whether fat in this depot could exert local anti-atherosclerosis effects, however, are not defined in this study and need further exploration.

In summary, acquired generalized lipodystrophy by surgical fat removal exacerbated metabolic disorders but not atherogenesis in LDLR^−/−^ mice fed on HFD.

## Methods

### Animals and diets

Eighteen male LDLR^−/−^ mice were purchased from Vital River Laboratories (Beijing, China) and maintained on a 12 h light/12 h dark cycle at 24 °C, with free access to water and standard laboratory chow diet. The Principles of Laboratory Animal Care (NIH publication no.85Y23, revised 1996) was followed, and the experimental protocol was approved by the Animal Care Committee, Hebei Medical University.

When mice were 16–20 weeks old, they were classified into Sham group and Fat-removed group. Fat removal surgery was performed after the mice were anesthetized with 1% pentobarbital sodium and lost their pedal and corneal reflexes. Placed face-up, the mice were subjected to a midventral abdominal incision. Bilateral subcutaneous fats in the inguinal were exposed, removed and weighed. Then, the abdominal cavity was opened. Bilateral epididymal fats were exposed, removed and weighted. After the peritoneum and the skin were sutured, mice were placed back side up, bilateral scapula brown fats were exposed, removed and weighed. Relative fat pads in the sham-operated control group were mobilized without removed.

One week after the surgery, basic plasma glucose and lipids were determined and glucose tolerance tests (GTT) were implemented. Then, mice were fed on an atherogenic high-fat diet (HFD, consisting of 20% fat and 0.5% cholesterol)^26^ for the next 12 weeks. Body weight and plasma lipids were monitored during HFD feeding.

After 12 weeks on the HFD, mice were anesthetized and washed with 20 mL 10 µM phosphate-buffered saline (PBS) through left ventricle. Then tissues were harvested. The heart, liver, aorta, retroperitoneal fat and mesenteric fat were collected for further analysis. Tissues for subsequent Western-blot or real-time PCR were flash-frozen in liquid nitrogen and then kept at −80 °C until they were analyzed, whereas tissues for histology were fixed in 4% PFA for 6–12 h at 25 °C, then stored at 4 °C in 20% sucrose until analysis.

### Plasma biochemical characteristics

Plasma was gained by retro-orbital bleeding. The levels of plasma total cholesterol (TC), triglyceride (TG) and glucose (GLU) were estimated by enzymatic methods (Bio Sino, Beijing, China). As for the analysis of the High-density lipoprotein cholesterol (HDL-C), after the ApoB-lipoprotein was formed sediment with 20% polyethylene glycol solution, HDL-C was determined with the TC kit. Enzymatic methods (Wako, Japan) was used when non-esterified fatty acid (NEFA) was measured. While ELISA kit (Ex cell, China) was used for the determination of insulin.

### Glucose and insulin tolerance tests

All the experimental animal were fasted for 4 hours when performed glucose and insulin tolerance tests, followed by being subjected with i.p. glucose (2 g/kg body weight; Abbott) or insulin ((0.75 mIU/g body weight; Humulin). Blood specimens were gathered before (time 0) and at 15, 30, 60 and 120 (for GTT) or 90 (for ITT) minutes after the intraperitoneal injection. Enzymatic methods was used when the levels of plasma glucose were determined as what we had described above.

### Histological studies

The adipose tissues and livers were paraffin-embedded after fixation, sectioned at 7 μm thickness and stained by hematoxylin/eosin staining (H&E). The quantification of the adipocytes was performed by Image J software. After fixed, the liver was also performed by OCT-embedded (OCT: Sakura Finetek, USA). We obtained sections with a thickness of 7 μm by cryo-sectioning. Lipid deposition was determined by Oil-red O staining of the cyro-sections of the liver.

For atherosclerosis analysis, after being visualized by Oil-red O staining, lesion area in *en face* aorta was then measured by Image J Graphic Analysis System. The quantification of the aortic root was performed on aorta cross sections as what we had previously described^27^. Briefly, we used OCT to embed the heart, and then put it in liquid nitrogen for making it snap-frozen and stored it at −30 °C before sectioning. 7 μm serial sections were made from the origin of the aorta in which all three aortic apex were clearly observed. Atherosclerotic lesion was visualized by Oil-red O staining followed by nuclear staining with hematoxylin and quantified by the average Oil-red O^+^ area per 8 sections for each mouse with Image J. For the necrotic core analysis, H&E was performed for the stainings on sections, and Sirius red was applied for fibrosis analysis. As for immuno-detection, CD68 antibody was used for macrophage, while SM22α was used for smooth muscle cells (Santa Cruz Biotechnology, Dallas, TX).

### RNA isolation and quantitative real-time PCR

To extract the total RNA from the adipose tissue or liver, Trizol reagent (Invitrogen, USA) was used. In order to generate first-strand cDNA, a RT kit (Invitrogen, USA) was used, and the produced cDNA was performed quantitative real-time PCR by using primers listed in Supplementary Dataset 1. Amplifications were carried out in 35 cycles applying an Applied Biosystems and Quant Studio™ Design & Analysis Software, with SYBR green fluorescence (TransGene Biotech, Beijing, China). We quantitated all of the samples by the comparative CT method for the purpose of relative quantitation of gene expression, and relative quantitation was normalized to GAPDH.

### Western blot analysis

Tissues were put into RIPA buffer and then were homogenized. For the protein content measuring, a bicinchoninic acid protein assay kit (Pierce, Rockford, IL) was used. After being loaded, identical amount of proteins were separated by SDS-polyacrylamide gel electrophoresis. After being transferred on nitrocellulose membranes (Applygen Technologies, Beijing, China), the proteins were identified with the required primary antibodies. Then the protein-antibody complex were recognized with secondary antibodies which were conjugated to horseradish peroxidase. As for the development of the blots, the enhanced chemi-luminescence detection reagents (ThermoFisher Scientific, Shanghai, China) was applied. Primary antibodies used in the study includes Akt, phospho-Akt (Ser473) (Cell signaling technology, Danvers, MA).

### Liver lipid analysis

As for the liver lipid analysis, we weighed approximately 100 mg of liver (wet weight) and homogenized it in 1 mL PBS. Lipids were extracted by chloroform-methanol (2:1, V/V) and dissolved in 0.5 mL 3% Triton X-100. Enzymatic methods was used as what we described above to analyze the content of triglyceride and cholesterol.

### Statistical analysis

All of the data were shown as means ± SEM. And statistics was performed using the student’s *t* test with GraphPad Prism 5.0. P value < 0.05 was considered statistically significant.

## Supplementary information

Dataset 1

Dataset 2
