# Author Correction: Surgical fat removal exacerbates metabolic disorders but not atherogenesis in LDLR^−/−^ mice fed on high-fat diet

**DOI:** 10.1038/s41598-020-58201-5

**Published:** 2020-02-04

**Authors:** Lin Liu, Chenxi Liang, Xiaowei Wang, Xiayu Ding, Yingjing Lu, Jinghui Dong, Mei Han, Hongyuan Yang, Mingming Gao, Jiawei Liao

**Affiliations:** 1 Laboratory of Lipid Metabolism, Institute of Basic Medicine, Hebei Medical University, Shijiazhuang, Hebei 050017 China; 2 Department of Physiology, Hebei Medical University, Shijiazhuang, Hebei 050017 China; 3 Department of Biochemistry and Molecular Biology, College of Basic Medicine, Key Laboratory of Medical Biotechnology of Hebei Province, Hebei Medical University, Shijiazhuang, Hebei 050017 China; 4 School of Biotechnology and Biomolecular Sciences, The University of New South Wales, Sydney, NSW 2052 Australia; 5 Department of Cardiology, Institute of Cardiovascular Diseases, First Affiliated Hospital of Dalian Medical University, Dalian, Liaoning 116011 China

Correction to: *Scientific Reports* 10.1038/s41598-019-54392-8, published online 28 November 2019

The original version of this Article contained an error in the order of author names, which were incorrectly given as ‘Lin Liu, Chenxi Liang, Xiaowei Wang, Xiayu Ding, Yingjing Lu, Jinghui Dong, Mei Han, Hongyuan Yang, Jiawei Liao & Mingming Gao’.

This has now been corrected in the HTML and PDF versions of this Article, and in the accompanying Supplementary Information files.

