# Supplementary material for: Surgical fat removal exacerbates metabolic disorders but not atherogenesis in LDLR−/− mice fed on high-fat diet
Source: Sci Rep. 2019 Nov 28;9:17848. doi: 10.1038/s41598-019-54392-8 (PMC6883051; doi:10.1038/s41598-019-54392-8)
Supplement: Supplementary file 1 — Dataset 1 [file 41598_2019_54392_MOESM1_ESM.pdf]

Surgical fat removal exacerbates metabolic disorders but not atherogenesis in LDLR$^{-/-}$ mice fed on high-fat diet

Lin Liu[1], Chenxi Liang[1], Xiaowei Wang[1], Xiayu Ding[1], Yingjing Lu[1], Jinghui Dong[2], Mei Han[3], Hongyuan Yang[4], Jiawei Liao[5,*], Mingming Gao[1,*]

[1] Laboratory of Lipid Metabolism, Institute of Basic Medicine, Hebei Medical University, Shijiazhuang, Hebei 050017, China

[2] Department of Physiology, Hebei Medical University, Shijiazhuang, Hebei 050017, China

[3] Department of Biochemistry and Molecular Biology, College of Basic Medicine, Key Laboratory of Medical Biotechnology of Hebei Province, Hebei Medical University, Shijiazhuang, Hebei 050017, China

[4] School of Biotechnology and Biomolecular Sciences, the University of New South Wales, Sydney, NSW, 2052, Australia

[5] Department of Cardiology, Institute of Cardiovascular Diseases, First Affiliated Hospital of Dalian Medical University, Dalian, Liaoning 116011, China.

*To whom correspondence should be addressed. Email: g.m0515@163.com, liaojiawei@bjmu.edu.cn,

Tel: +86-311-86261102; Fax: +86-311-86261102.

Table 1: A list of primers for qPCR

Gene	Forward	Reverse
Gapdh	TGATGACATCAAGAAGGTGGTGAAG	TCCTTGGAGGCCATGTAGGCCAT
Pparg	GACCACTCGCATTCCTTT	CCACAGACTCGGCACTCA
Cebpa	GTTAGCCATGTGGTAGGAGACA	CCCAGCCGTTAGTGAAGAGT
Fasn	GGGTCTATGCCACGATTC	GTGTCCCATGTTGGATTTG
Acc1	CTCCCGATTCATAATTGGGTCTG	TCGACCTTGTTTTACTAGGTGC
Scd1	CGCTGGCACATCAACTTCAC	AGGAACTCAGAAGCCCAAAGC
Dgat2	GCGCTACTTCCGAGACTACTT	GGGCCTTATGCCAGGAAACT
Atgl	ATGTTCCCGAGGGAGACCAA	GAGGCTCCGTAGATGTGAGTG
Hsl	GATTTACGCACGATGACACAGT	ACCTGCAAAGACATTAGACAGC
Cd36	GGAGCCATCTTTGAGCCTTCA	GAACCAAACTGAGGAATGGATCT
Ppara	GGGCTTTCGGGATAGTTG	ATTGGGCTGTTGGCTGAT
Pgc1a	TATGGAGTGACATAGAGTGTGCT	GTCGCTACACCACTTCAATCC
Acox1	GTACCAGCGTCGGGGATTG	AAAGGCTCAGGATGCCCTCG
Cpt1a	CTCCGCCTGAGCCATGAAG	CACCAGTGATGATGCCATTCT
Mttp	ATACAAGCTCACGTACTCCACT	TCTCTGTTGACCCGCATTTTC
Chrebp	AGCATCGATCCGACACTCAC	TTGTTCAGCCGGATCTTGTC
Lxr	GCGACAGTTTTGGTAGAGGGAC	CGCTTTTGTGGACGAAGCTC
Npc1	CTGTGACCTGATCCCTACCC	CCTGTCTTCCCGGGCCATAA
Lrp1	CTCCCACCGCTATGTGATCC	CACAGCTGTTGGTGTCGTTG
Srb1	CGAAGTGGTCAACCCAAACGA	CCATGCGACTTGTCAGGCT
Abca1	AAAACCGCAGACATCCTTCAG	CATACCGAAACTCGTTCACCC
Acat1	CAGGAAGTAAGATGCCTGGAAC	TGCAGCAGTACCAAGTTTAGTG
