# Supplementary material for: Surgical fat removal exacerbates metabolic disorders but not atherogenesis in LDLR−/− mice fed on high-fat diet
Source: Sci Rep. 2019 Nov 28;9:17848. doi: 10.1038/s41598-019-54392-8 (PMC6883051; doi:10.1038/s41598-019-54392-8)

Surgical fat removal exacerbates metabolic disorders but not atherogenesis in LDLR$^{-/-}$ mice fed on high-fat diet

Lin Liu[1], Chenxi Liang[1], Xiaowei Wang[1], Xiayu Ding[1], Yingjing Lu[1], Jinghui Dong[2], Mei Han[3], Hongyuan Yang[4], Jiawei Liao[5,]*, Mingming Gao[1,] *

[1] Laboratory of Lipid Metabolism, Institute of Basic Medicine, Hebei Medical University, Shijiazhuang, Hebei 050017, China

[2] Department of Physiology, Hebei Medical University, Shijiazhuang, Hebei 050017, China

[3] Department of Biochemistry and Molecular Biology, College of Basic Medicine, Key Laboratory of Medical Biotechnology of Hebei Province, Hebei Medical University, Shijiazhuang, Hebei 050017, China

[4] School of Biotechnology and Biomolecular Sciences, the University of New South Wales, Sydney, NSW, 2052, Australia

[5] Department of Cardiology, Institute of Cardiovascular Diseases, First Affiliated Hospital of Dalian Medical University, Dalian, Liaoning 116011, China.

*To whom correspondence should be addressed. Email: g.m0515@163.com, liaojiawei@bjmu.edu.cn,

Tel: +86-311-86261102; Fax: +86-311-86261102.

Figure 2f

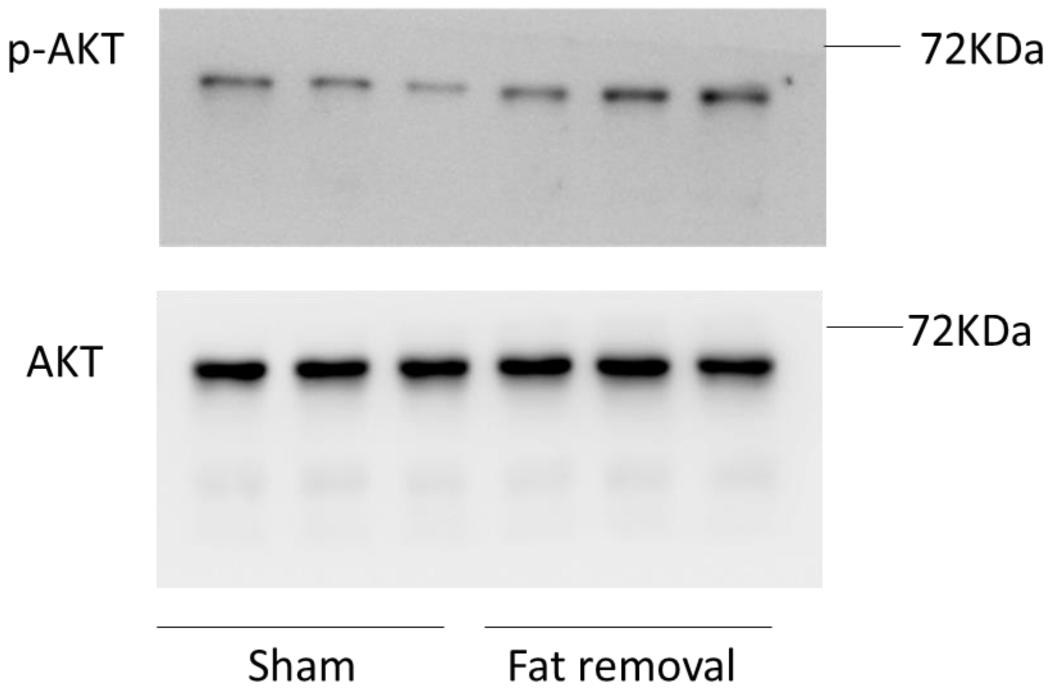

Figure 2g

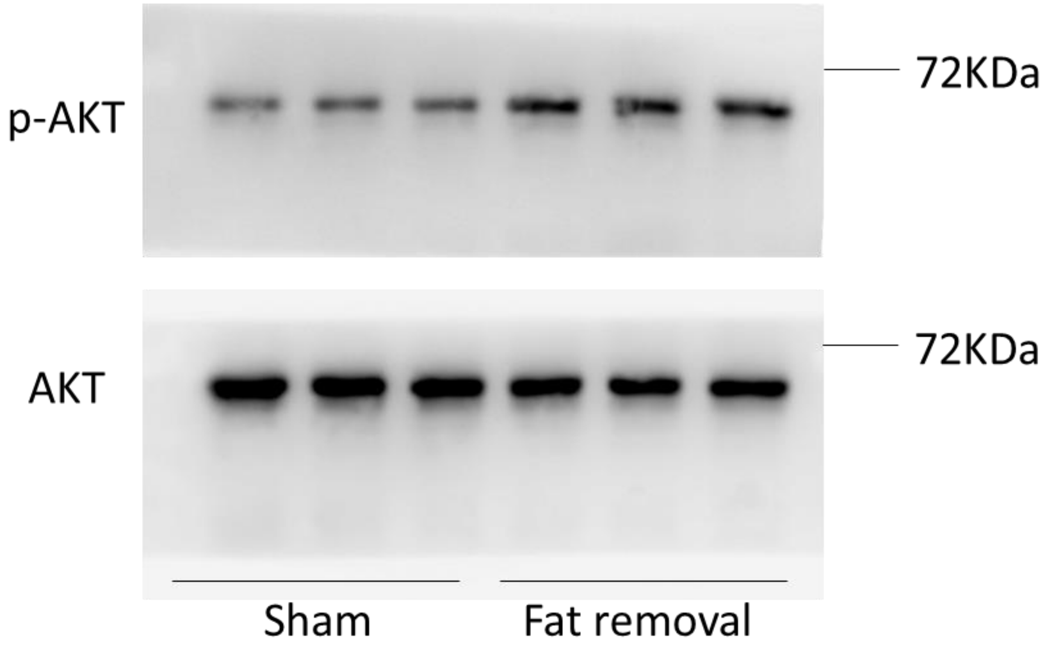

Figure 4i

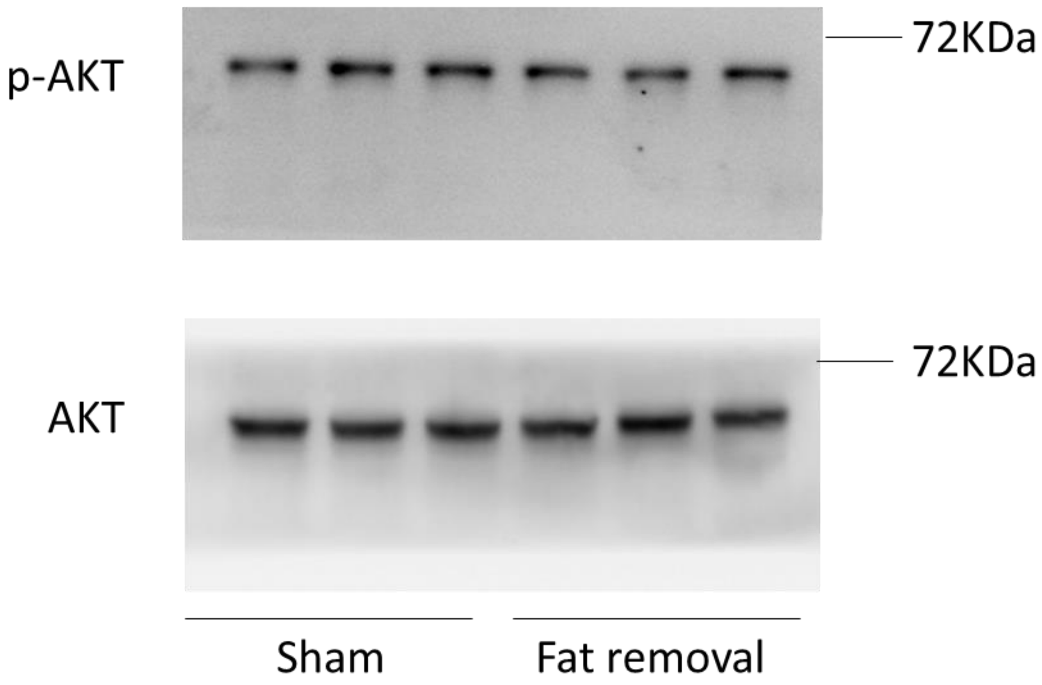

Figure 4j

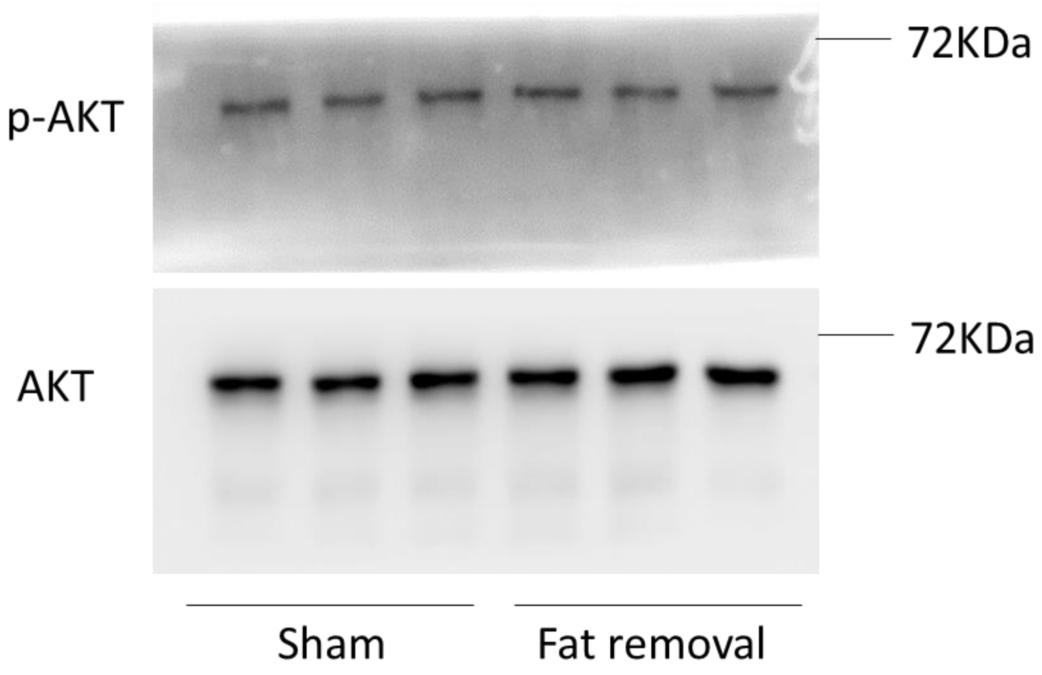

Supplement: Supplementary file 2 — Dataset 2 [file 41598_2019_54392_MOESM2_ESM.pdf]
